# Circ_0043532 regulates miR-182/SGK3 axis to promote granulosa cell progression in polycystic ovary syndrome

**DOI:** 10.1186/s12958-021-00839-5

**Published:** 2021-11-05

**Authors:** Lishuang Xu, Fang Xiong, Yinyang Bai, Juxia Xiao, Yun Zhang, Jie Chen, Qiuping Li

**Affiliations:** Department of Center of Reproductive Medicine, The Affiliated Wuxi Maternity and Child Health Care Hospital of Nanjing Medical University, NO.48 Huaishu Street, Wuxi, 214002 Jiangsu China

**Keywords:** PCOS, circ_0043532, miR-182, SGK3

## Abstract

**Background:**

Polycystic ovary syndrome (PCOS) is a common endocrine and metabolic disease in women at childbearing age. Several circular RNAs (circRNAs) have been demonstrated to be involved in PCOS. In this study, we aimed to explore the function and mechanism of circ_0043532 in PCOS.

**Methods:**

Quantitative real-time polymerase chain reaction (qRT-PCR) was performed to determine the expression of circ_0043532, miR-182 and serum/glucocorticoid regulated kinase family member 3 (SGK3). Cell proliferation was assessed by 5-ethynyl-2′-deoxyuridine (EdU) assay and 3-(4, 5-dimethyl-2-thiazolyl)-2, 5-diphenyl-2-H-tetrazolium bromide (MTT) assay. Flow cytometry analysis was employed to evaluate cell cycle and cell apoptosis. Dual-luciferase reporter assay and RNA immunoprecipitation (RIP) assay were conducted to verify the association between miR-182 and SGK3. Western blot assay was carried out to determine the protein level of SGK3.

**Results:**

Circ_0043532 was markedly elevated in PCOS granulosa cells (GCs) and KGN cells. Silencing of circ_0043532 suppressed cell proliferation and cell cycle process and promoted cell apoptosis in PCOS GCs and KGN cells. For mechanistic analysis, circ_0043532 was identified as a sponge of miR-182 and SGK3 was confirmed to be a target gene of miR-182. Inhibition of miR-182 rescued the impacts of circ_0043532 interference on PCOS GCs and KGN cell progression. Moreover, miR-182 overexpression suppressed cell proliferation and cell cycle process and promoted cell apoptosis in PCOS GCs and KGN cells by targeting SGK3.

**Conclusion:**

Deficiency of circ_0043532 suppressed cell proliferation and induced cell cycle arrest and cell apoptosis in PCOS by modulation of miR-182/SGK3 axis.

## Highlights

1. Circ_0043532 and SGK3 are increased and miR-182 is decreased in PCOS granulosa cells and KGN cells.
2. Circ_0043532 knockdown represses cell proliferation and induces cell apoptosis and cell cycle arrest in G0-G1 phase in PCOS granulosa cells and KGN cells.
3. Circ_0043532 positively regulates SGK3 expression by targeting miR-182 in PCOS granulosa cells and KGN cells.
4. Circ_0043532 regulates the development of PCOS through regulating miR-182/SGK3 axis.

## Introduction

Polycystic ovary syndrome (PCOS) is a common endocrine disease in reproductive-aged females, affecting 6% ~ 10% of women in the world [1]. It is characterized by chronic anovulation, hyperandrogenism, polycystic ovary and metabolic disorders [2]. The typical clinical manifestations of PCOS including abnormal menstruation, infertility, hairy, acne, and obesity [3]. More than 20% of women with infertility problems are associated with PCOS [4]. However, the etiology of PCOS is very complicated. Hence, it is very necessary to elucidate the pathogenesis and underlying mechanism of PCOS.

Circular RNAs (circRNAs) are a family of non-coding RNAs (ncRNAs) with covalently closed loop structure [5]. Presently, only very few circRNAs have been identifed to be aberrantly expressed in PCOS and participate in regulating PCOS progression. For example, Jia et al. claimed that circ_0118530 was increased PCOS patients and granulosa cells (GCs) and circ_0118530 knockdown suppressed cell viability and migration and facilitated apoptosis in KGN cells by sponging miR-136 [6]. Zhao et al. disclosed that the elevation of circ_0023942 repressed the growth of ovarian GCs [7]. Moreover, Ma et al. demonstrated that circ_0043532 was abnormally elevated in PCOS patients [8]. Nonetheless, the precise roles of circ_0043532 in PCOS progression are barely known.

MicroRNAs (miRNAs), small ncRNAss with about 22 nucleotides, function as gene regulators via recognizing the 3′-untranslated region (3′ UTR) of target mRNAs [9]. It has been documented that the aberrant expression of miRNAs was associated with some biological processes, such as cell proliferation, metastasis, differentiation and apoptosis [10]. In recent, more and more miRNAs have been confirmed to be associated with the progression of diverse human diseases, including PCOS [11, 12]. For example, Cai et al. implicated that miR-145 was reduced in PCOS GCs and miR-145 overexpression repressed cell growth and induced cell apoptosis in PCOS GCs via targeting IRS1 [13]. Xiang et al. proved that there was a decrease of miR-483 in ovarian cortexes from patients with PCOS, and miR-483 could hamper KGN cell growth and induce cell cycle arrest via interacting with IGF1 [14]. However, Jiang et al. disclosed that miR-93 was elevated in PCOS ovarian cortex tissues and the elevated expression of miR-93 facilitated GCs growth and cell cycle process in PCOS through binding to CDKN1A [15]. These reports indicated that miRNAs play different roles in PCOS. MiR-182 has been revealed to be decreased in granulosa-lutein cells from PCOS patients [16]. Nevertheless, the exact function and mechanism of miR-182 in PCOS remain unknown.

Serum/glucocorticoid regulated kinase family member 3 (SGK3), a member of Aggrecan (AGC) protein kinase family member, acts as an oncogene in human cancers such as osteosarcoma (OS) [17], hepatocellular carcinoma (HCC) [18] and breast cancer [19]. Moreover, SGK3 was highly expressed in PCOS GCs and participated in the development of PCOS [20]. However, the reports on SGK3 in PCOS are still very few and whether SGK3 could be a target of miR-182 remains unclear.

Here, we determined the expression of circ_0043532, miR-182 and SGK3 in PCOS GCs and KGN cells. Besides, their functions and underlying mechanisms in cell proliferation, cell cycle and apoptosis were explored.

## Materials and methods

### Clinical samples and cell culture

Thirty PCOS patients (without endometriosis) who underwent laparoscopic investigation for infertility and 10 volunteers who underwent diagnostic laparoscopy or laparoscopic disinfection for pelvic pain at The Affiliated Wuxi Maternity and Child Health Care Hospital of Nanjing Medical University were enrolled in our study. PCOS was diagnosed according to the criteria: (1) chronic oligovulatory and/or anovulation (cycle length < 26 days or > 35 days); (2) Clinical hyperandrogenemia and biochemical hyperandrogenemia; (3) Ultrasound examination of polycystic ovary. The study was permitted by the Ethics Committee of The Affiliated Wuxi Maternity and Child Health Care Hospital of Nanjing Medical University. Written informed consents were provided by all participants.The correlation between circ_0043532 or miR-182 expression and clinicopathologic features of PCOS patients were exhibited in Table 1.

**Table 1:** Correlation between circ_0043532 or miR-182 expression and clinicopathologic features of PCOS patients

Clinicopathologic features	Relative circ_0043532 level		*P* value	Relative miR-182 level		*P* value
	High	Low		Low (%)	High (%)	
Age (years)			0.7651			0.654
≥ 30	10	8		9	9	
< 30	6	6		7	5	
BMI (kg/m^2^)			0.5104			0.3894
20–26	5	6		7	4	
< 20 or > 26	11	8		9	10	
Basal LH/FSH			0.232			0.654
> 3	8	10		9	9	
2–3	8	4		7	5	
Testosterone (ng/ml)			0.3508			0.0935
> 1	3	1		2	2	
0.5–1	13	13		14	12	

GCs were isolated from the follicular fluid of all participants as previously described [21]. The human granulosa-like tumor cell line KGN was bought from Riken Cell Bank (Riken Institute, Japan) and normal ovarian epithelial cell line IOSE80 was bought from ScienCell Research Laboratories (San Diego, CA, USA). All cells were cultured in Dulbecco’s modified Eagle’s medium (DMEM)/F12 medium (Invitrogen, Carlsbad, CA, USA) including 10% fetal bovine serum (FBS; Invitrogen) and 1% penicillin-streptomycin (Invitrogen) at an atmosphere of 5% CO_2_ and 37 °C.

### Cell transfection

Small interfering RNA targeting circ_0043532 (si-circ_0043532; 5′-AAACATTGCAACCCAGCTGTT-3′) and its control (si-con; 5′-TTCTCCGAACGTGTCACGTTT-3′), mimics of miR-182 (miR-182; 5′-TTTGGCAATGGTAGAACTCACACT-3′) and its control (miR-con; 5′-TTCTCCGAACGTGTCACGTTT-3′), inhibitors of miR-182 (in-miR-182; 5′-TTCTACCATTGCCAA-3′) and its control (in-miR-con; 5′-CAGTACTTTTGTGTAGTACAA-3′), small interfering RNA against SGK3 (si-SGK3; 5′-GAGAGTAACTACAGAAGAACTTT-3′) and its control (si-con; 5′-TTCTCCGAACGTGTCACGTTT-3′); the overexpression plasmid of SGK3 (SGK3) and its control (pcDNA) were all synthesized by GeneCopoeia (Guangzhou, China). Then GCs and KGN (1 × 10^5^ cells/well) were plated to 24-well plates and transfected with 50 nM synthesized oligonucleotides or 2 μg synthesized vectors through the usage of Lipofectamine 2000 (Invitrogen) according to the manufacturers’ instructions.

### Quantitative real-time polymerase chain reaction (qRT-PCR)

Total RNA in GCs, KGN and IOSE80 cells was extracted from using TRIzol reagent (Invitrogen) and examined on a NanoDrop 2000c spectrophotometer (Thermo Fisher Scientific, Waltham, MA, USA). Then complementary DNA (cDNA) was synthesized using miRNA 1st Strand cDNA Synthesis Kit (Vazyme, Nanjing, China) or M-MLV First Strand Kit (Takara, Dalian, China). Afterward, qRT-PCR was conducted using AceQ Universal SYBR qPCR Master Mix (Vazyme) on an ABI 7900 Real-Time PCR system (Applied Biosystems, Foster City, CA, USA) under the thermocycling conditions: initial denaturation at 95 °C for 5 min; 40 cycles of 95 °C for 10 s and 60 °C for 30 s; 95 °C for 15 s, 60 °C for 60 s and 95 °C for 15 s. The expression levels of miR-182 and SGK3 were measured using the 2^-ΔΔCt^ method [22]. Glyceraldehyde 3-phosphate dehydrogenase (GAPDH) or small nuclear RNA U6 was used as an internal reference. The primers were: circ_0043532: (F: 5′-TGTTCTAAACATTGCAACCCAGC-3′ and R: 5′-AGGCAAAACTTCAGCCATTTGT-3′); miR-182: (F: 5′-GCTTTGGCAATGGTAGAACT-3′ and R: 5′-GAACATGTCTGCGTATCTC-3′); SGK3: (F: 5′-CCAGGAGTGAGTCTTACAG-3′ and R: 5′-CCAGCCACATTAGGATTA-3′); GAPDH: (F: 5′-TGTTCGTCATGGGTGTGAAC-3′ and R: 5′-ATGGCATGGACTGTGGTCAT-3′); U6: (F: 5′-ATTGGAACGATACAGAGAAGATT-3′ and R: 5′-GGAACGCTTCACGAATTTG-3′).

### 5-ethynyl-2′-deoxyuridine (EdU) assay

PCOS GCs and KGN cells (1 × 10^4^ cells/well) were cultured in 24-well plates for 48 h. Next, the cells were interacted with EdU (Beyotime, Shanghai, China), fixed in paraformaldehyde (Sigma-Aldrich, St. Louis, MO, USA), mixed with 0.5% Triton-X-100 (Sigma-Aldrich) and stained using DAPI (Sigma-Aldrich). The images were captured using a fluorescence microscope (Olympus, Tokyo, Japan) and EDU-positive cells were quantified.

### 3-(4, 5-dimethyl-2-thiazolyl)-2, 5-diphenyl-2-H-tetrazolium bromide (MTT) assay

After being transfected with indicated synthetic oligonucleotides or vectors, PCOS GCs and KGN cells (2 × 10^3^ cells/well) were seeded into 96-well plates. Then, 20 μL MTT (Solarbio, Beijing, China) was added to the well at indicated time points. After incubation for 4 h, 150 μL dimethylsulfoxide (DMSO; Solarbio) was added to the well to dissolve the formazan crystals. Finally, a microplate reader was used for assessing the optical density at 490 nm.

### Flow cytometry analysis

Cell cycle and cell apoptosis were evaluated using Annexin V-fluorescein isothiocyanate (FITC)/propidium iodide (PI) Apoptosis Detection Kit (Beyotime). For cell cycle analysis, transfected PCOS GCs and KGN cells were collected and fixed in ice-cold 75% ethanol overnight at 4 °C. Next, cells were washed, resuspended (2 × 10^5^ cells/mL) and incubated with PI (Beyotime) and RNase (Solarbio) for 30 min at room temperature. The cells were then analyzed using a flow cytometer (BD Biosciences, San Jose, CA, USA). For cell apoptosis analysis, transfected PCOS GCs and KGN cells were harvested, washed, resuspended and the incubated with 5 μL AnnexinV-FITC (Beyotime) and 10 μL PI (Beyotime) for 15 min to stain cells in the dark. Finally, cell apoptosis was assessed with a flow cytometry (BD Biosciences).

### Dual-luciferase reporter assay

The sequences of SGK3 containing the potential binding sites of wild-type or mutant miR-182 were introduced into pmirGLO vector (Promega, Madison, WI, USA) to establish the luciferase vectors SGK3-WT and SGK3-MUT, respectively. Then the vector was transfected into PCOS GCs and KGN cells together with miR-182, miR-con, in-miR-con or in-miR-182 using Lipofectamine 2000 (Invitrogen). After 48 h, the luciferase activity was measured using Dual-Luciferase Reporter Assay Kit (Promega). The renilla luciferase activity was normalized to firefly luciferase activity.

### RNA immunoprecipitation (RIP) assay

Magna RNA-binding protein immunoprecipitation kit (EMD Millipore, Billerica, MA, USA) was utilized to conducted RIP assay. In brief, PCOS GCs and KGN cells were lysed in RIP buffer and then cell extracts (100 mL) were incubated with magnetic beads which were coated with Anti-Argonaute2 (Anti-Ago2; Abcam, Cambridge, MA, USA) or immunoglobulin G (Anti-IgG; Abcam) for 8 h at 4 °C. Afterward, samples were digested by Proteinase K (Solarbio) for 30 min. Finally, RNAs in the complex were isolated and then qRT-PCR was conducted to evaluate the enrichment of SGK3 and miR-182.

### Western blot assay

Total protein was isolated from cells with RIPA buffer (Beyotime) and measured on a NanoDrop 2000c spectrophotometer (Thermo Fisher Scientific). After being separated by sodium dodecyl sulfonate-polyacrylamide gel (SDS-PAGE; Solarbio), the same amount of proteins (30 μg) were transferred onto polyvinylidene difluoride membranes (PVDF; EMD Millipore). Next, the membranes were blocked with 5% slim milk in TBST for 2 h and incubated with primary antibody: anti-SGK3 (ab153981; Abcam), anti-BAX (ab53154; Abcam) or β-actin (ab8226; Abcam) overnight at 4 °C. After that, the membranes were washed with TBST for 3 times and incubated with relevant secondary antibody (ab150117; Abcam) for 2 h at room temperature. The protein bands were analyzed using an enhanced chemiluminescence reagent (EMD Millipore) and analyzed using ImageJ software (NIH, Bethesda, MD, USA).

### Statistical analysis

The data collected from three independent experiments were analyzed using software GraphPad Prism 7 (GraphPad Inc., La Jolla, CA, USA) and displayed as mean ± standard deviation (SD). Different analysis was performed using Student’s *t*-test or one-way Analysis of Variance (ANOVA). The correlations among circ_0043532, miR-182 and SGK3 in PCOS GCs was analyzed by Spearman’s correlation coefficient analysis. *P* < 0.05 was defined to be a significant difference.

## Results

### Knockdown of circ_0043532 suppressed cell proliferation and cell cycle process and promoted cell apoptosis in PCOS GCs and KGN cells

**Fig. 1:**
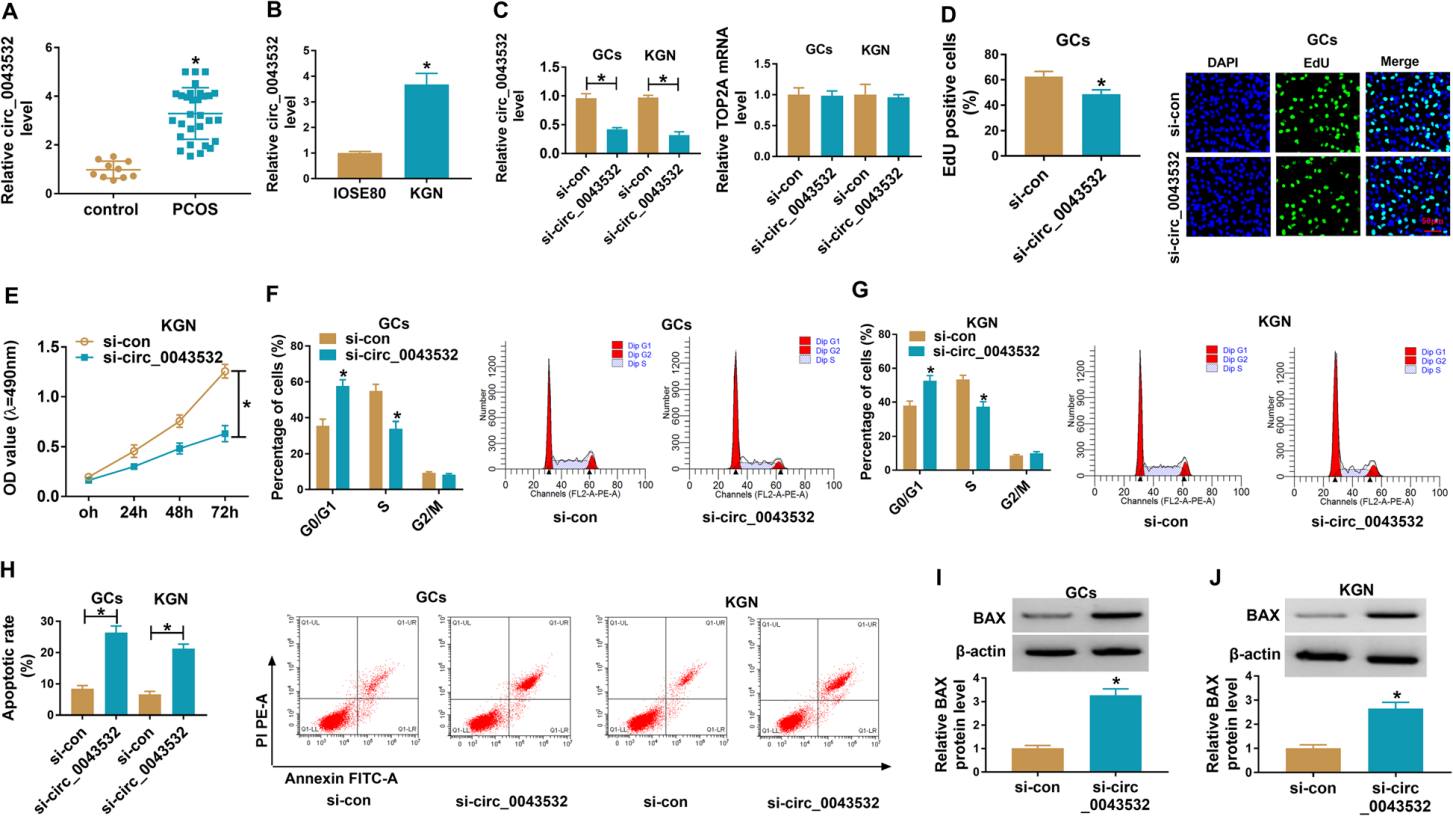
Circ_0043532 knockdown hampered cell proliferation and cell cycle process and facilitated cell apoptosis in PCOS GCs and KGN cells. (**A**) The expression of circ_0043532 in PCOS GCs and non-PCOS GCs was measured by qRT-PCR. (**B**) The expression of circ_0043532 in IOSE80 cells and KGN cells was measured by qRT-PCR analysis. (**C**-**H**) PCOS GCs and KGN cells were transfected with si-con or si-circ_0043532. (**C**) QRT-PCR assay was conducted for circ_0043532 and TOP2A expression in PCOS GCs and KGN cells. (**D** and **E**) The proliferation of PCOS GCs and KGN cells was determined via EdU assay and MTT assay. (**F**-**H**) Cell cycle and cell apoptosis in PCOS GCs and KGN cells were analyzed by flow cytometry analysis. (**I** and **J**) The protein level of BAX in PCOS GCs and KGN cells was measured via western blot assay. *P < 0.05

To begin with, qRT-PCR assay was conducted to determine the expression of circ_0043532 in GCs from 30 PCOS patients and 10 normal controls. As shown in Fig. 1A, circ_0043532 was highly expressed in GCs from PCOS patients compared to control groups. Moreover, we found that circ_0043532 was markedly upregulated in KGN cells compared to IOSE80 cells (Fig. 1B). These results indicated that the dysregulation of circ_0043532 might be associated in the progression of PCOS. Subsequently, to investigate the effect of circ_0043532 on PCOS progression, si- circ_0043532 was transfected into PCOS GCs and KGN cells to knockdown the expression of circ_0043532. The transfection efficiency was evaluated by qRT-PCR assay, showing that circ_0043532 was drastically declined in PCOS GCs and KGN cells transfected with si- circ_0043532, but TOP2A mRNA level was not affected (Fig. 1C). EdU assay and MTT assay indicated that circ_0043532 knockdown conspicuously suppressed cell proliferation in PCOS GCs and KGN cells compared to control group (Fig. 1D and E). Flow cytometry analysis showed that cell number in G0-G1 phase was distinctly increased, but cell number in S phase was distinctly decreased in PCOS GCs and KGN cells after circ_0043532 deficiency, indicting that circ_0043532 deficiency arrested cell cycle process (Fig. 1F and G). Flow cytometry analysis also showed that circ_0043532 silencing contributed to cell apoptosis in PCOS GCs and KGN cells compared to si-con groups (Fig. 1H). Moreover, circ_0043532 silencing increased BAX protein level in GCs and KGN cells (Fig. 1I and J). Collectively, circ_0043532 knockdown repressed cell proliferation and induced cell cycle arrest and cell apoptosis in PCOS GCs and KGN cells (Table 1).

### Circ_0043532 functioned as the sponge of miR-182

**Fig. 2:**
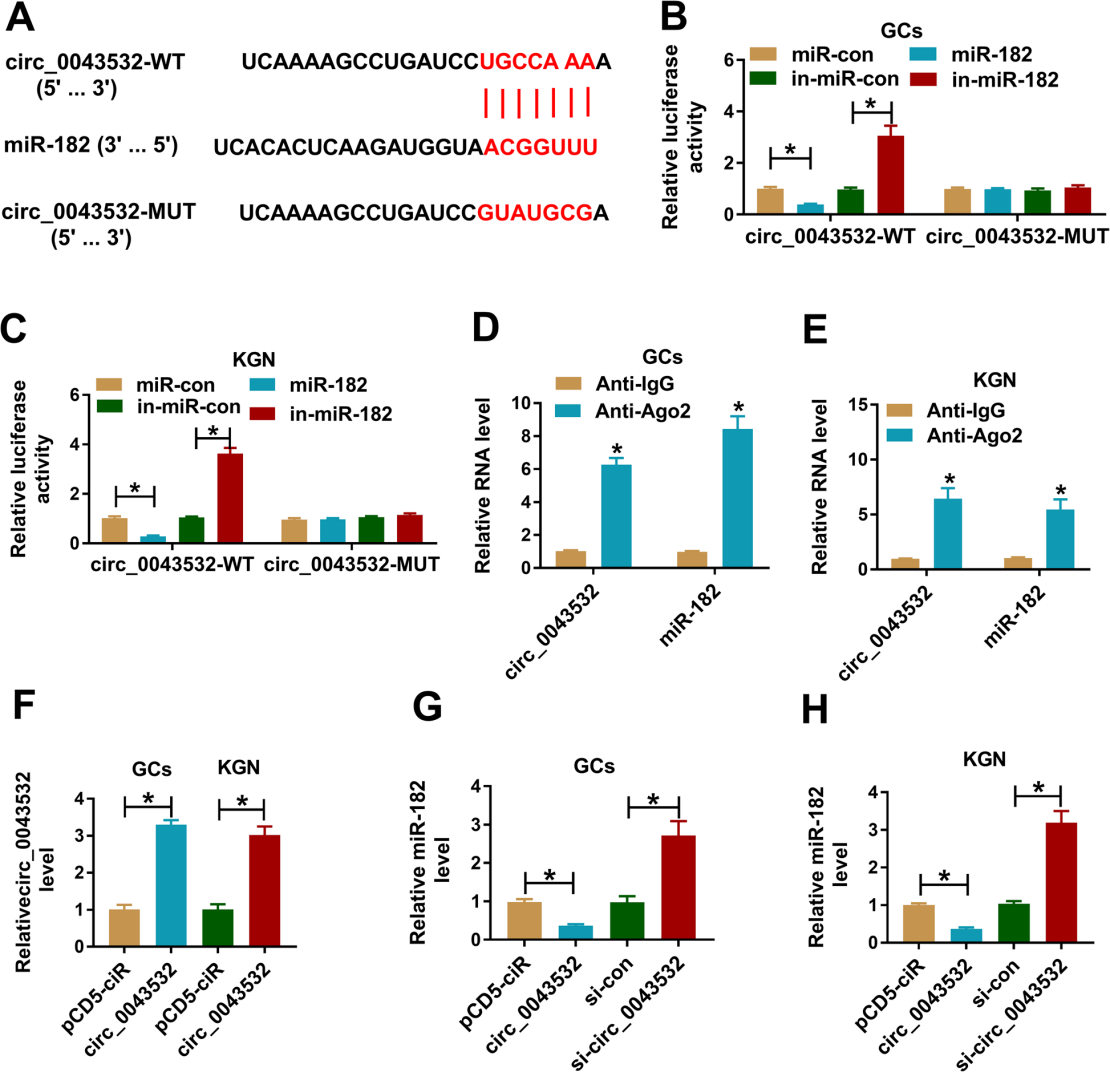
MiR-182 was targeted by circ_0043532. (**A**) The potential binding sites between miR-182 and circ_0043532. (**B** and **C**) The interaction between circ_0043532 and miR-182 was verified by dual-luciferase reporter assay. (**D** and **E**) The combination between circ_0043532 and miR-182 was confirmed by RIP assay. (**F**) The level of circ_0043532 in PCOS GCs and KGN cells transfected with pCD5-ciR or circ_0043532 was measured by qRT-PCR. (**G** and **H**) The level of miR-182 in PCOS GCs and KGN cells transfected with circ_0043532, pcDNA, si-circ_0043532 or si-con was determined by qRT-PCR assay. *P < 0.05

To explore the underlying mechanism of circ_0043532 in PCOS, online tool circular RNA Interactome was used to analyze the potential target of circ_0043532. The results showed that miR-182 contained the binding sties of circ_0043532 (Fig. 2A). Then dual-luciferase reporter assay and RIP assay were performed to verify the combination between circ_0043532 and miR-182. The results of dual-luciferase reporter assay exhibited that miR-182 drastically inhibited the luciferase activity of circ_0043532-WT and in-miR-182 obviously promoted the luciferase activity of circ_0043532-WT in PCOS GCs and KGN cells, while no changed was observed in circ_0043532-MUT groups (Fig. 2B and C). RIP assay showed that circ_0043532 and miR-182 were evidently enriched in Anti-Ago2 immunoprecipitation complex in PCOS GCs and KGN cells in relative to Anti-IgG control groups (Fig. 2D and E). As shown in Fig. 2F, circ_0043532 overexpression vector transfection led to a increase in circ_0043532 expression in PCOS GCs and KGN cells compared to pCD5-ciR control groups. Furthermore, we found that circ_0043532 overexpression distinctly decreased miR-182 level and circ_0043532 silencing apparently increased miR-182 level in both PCOS GCs and KGN cells (Fig. 2G and H). Taken together, circ_0043532 negatively modulated miR-182 expression by directly targeting.

### Inhibition of miR-182 restored the effects of circ_0043532 on cell proliferation, cell cycle and apoptosis in PCOS GCs and KGN cells

**Fig. 3:**
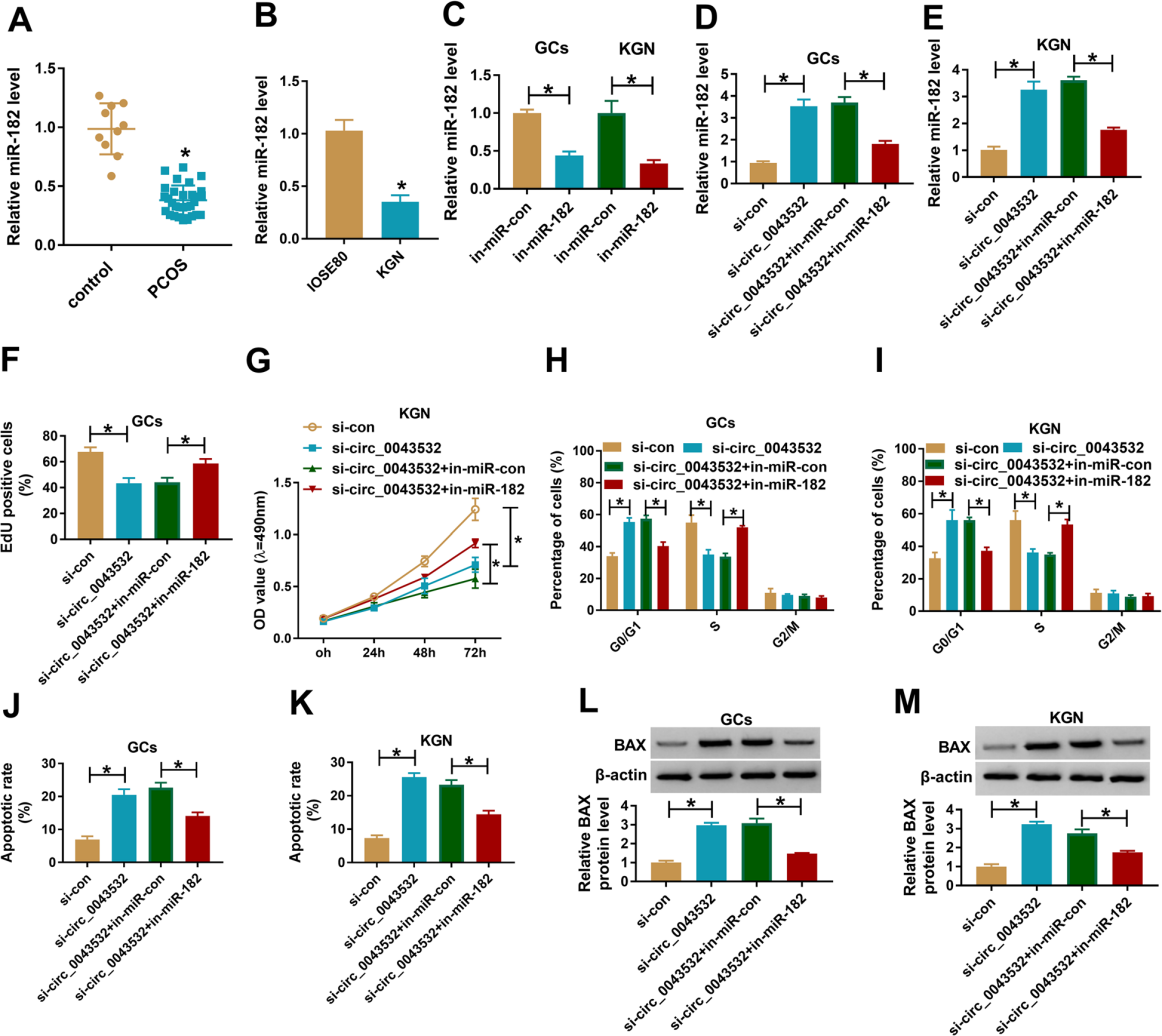
The effects of circ_0043532 knockdown on cell proliferation and promoted cell cycle arrest and apoptosis in PCOS GCs and KGN cells were reversed by miR-182 inhibition. (**A**) The expression of miR-182 in PCOS GCs and non-PCOS GCs was examined by qRT-PCR. (**B**) The expression of miR-182 in IOSE80 cells and KGN cells was measured with qRT-PCR. (**C**) The level of miR-182 in PCOS GCs and KGN cells transfected with in-miR-con or in-miR-182 was detected by qRT-PCR. (**D**-**M**) si-con, si-circ_0043532, si-circ_0043532 + in-miR-con or si-circ_0043532 + in-miR-182 was transfected into PCOS GCs and KGN cells. (**D** and **E**) The expression level of miR-182 in transfected PCOS GCs and KGN cells was examined through qRT-PCR analysis. (**F** and **G**) The proliferation of PCOS GCs and KGN cells was evaluated by EdU assay and MTT assay. (**H**-**K**) Cell cycle and cell apoptosis in PCOS GCs and KGN cells were analyzed by flow cytometry analysis. (**L** and **M**) The protein level of BAX in PCOS GCs and KGN cells was measured via western blot assay. *P < 0.05

Subsequently, we determined the expression level of miR-182 in PCOS GCs and KGN cells by qRT-PCR assay. The data showed that miR-182 was weakly expression in PCOS GCs and KGN cells in reference to normal GCs and IOSE80 cells (Fig. 3A and B). As shown in Fig. 3C, in-miR-182 transfection led to a reduction of miR-182 expression in PCOS GCs and KGN cells. Then PCOS GCs and KGN cells were transfected with si-con, si-circ_0043532, si-circ_0043532 + in-miR-con or si-circ_0043532 + in-miR-182 to explore the association between circ_0043532 and miR-182 in regulating PCOS development. As presented in Fig. 3D and E, the upregulation of miR-182 in PCOS GCs and KGN cells caused by circ_0043532 knockdown was reversed by the transfection of in-miR-182. Moreover, the inhibitory effects on cell proliferation and cell cycle and the promotional effect on cell apoptosis in PCOS GCs and KGN cells mediated by circ_0043532 knockdown was effectively rescued following the inhibition of miR-182 (Fig. 3F-K). Moreover, miR-182 inhibition reversed the effect of circ_0043532 knockdown on BAX protein level in PCOS GCs and KGN cells (Fig. 3L and M). Taken together, circ_0043532 knockdown decelerated the development of PCOS by targeting miR-182.

### SGK3 was a target gene of miR-182 in PCOS GCs and KGN cells

**Fig. 4:**
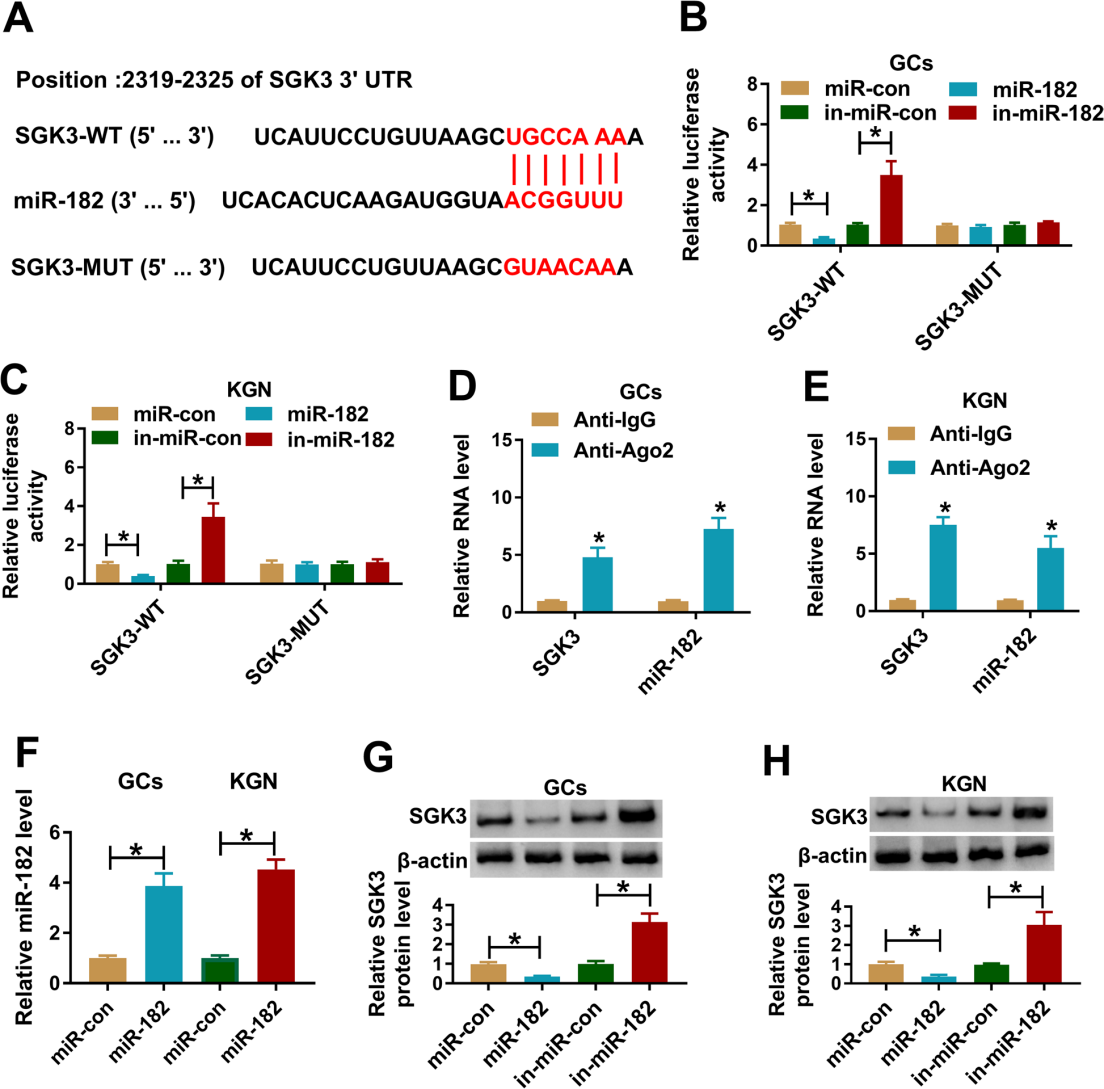
SGK3 was a target of miR-182 and negatively regulated by miR-182 in PCOS GCs and KGN cells. (**A**) The potential binding sites between miR-182 and SGK3 were exhibited. (**B** and **C**) PCOS GCs and KGN cells were co-transfected with SGK3-WT or SGK3-MUT and miR-con, miR-182, in-miR-con or in-miR-182 and then the luciferase activity was measured via dual-luciferase reporter assay. (**D** and **E**) The enrichment of SGK3 and miR-182 in Anti-Ago2 or Anti-IgG immunoprecipitation complex in PCOS GCs and KGN cells was detected by RIP and qRT-PCR assays. (**F**) The level of miR-182 in PCOS GCs and KGN cells transfected with miR-con or miR-182 was detected by qRT-PCR. (**G** and **H**) The protein level of SGK3 in PCOS GCs and KGN cells transfected with miR-con, miR-182, in-miR-con or in-miR-182 was examined by western blot assay. *P < 0.05

To further reveal the relationship between circ_0043532 and miR-182 in PCOS progression, online website TargetScan was utilized to predict the potential target of miR-182. The data showed that SGK3 was a target of miR-182 and their potential binding sites were exhibited in Fig. 4A. Then dual-luciferase reporter was conducted to verify this prediction. The data indicated that miR-182 and SGK3-WT co-transfection resulted in an obvious suppression, and in-miR-182 and SGK3-WT co-transfection led to a significant enhancement in the luciferase activity in PCOS GCs and KGN cells; however, the luciferase activity was not changed in SGK3-MUT group (Fig. 4B and C). RIP assay implicated that the levels of SGK3 and miR-182 were drastically increased in Anti-Ago2 immunoprecipitation complex in PCOS GCs and KGN cells compared to Anti-IgG control group (Fig. 4D and E). Besides, miR-con, miR-182, in-miR-con or in-miR-182 was transfected into PCOS GCs and KGN cells and then the expression level of SGK3 was measured by western blot assay. Our data showed miR-182 was successfully transfected into PCOS GCs and KGN cells, as demonstrated by qRT-PCR (Fig. 4F). Our data also manifested that miR-182 decreased SGK3 expression and in-miR-182 increased SGK3 expression in PCOS GCs and KGN cells (Fig. 4G and H). To sum up, miR-182 suppressed SGK3 expression by direct targeting in PCOS GCs and KGN cells.

### SGK3 overexpression attenuated the effects of miR-182 on cell proliferation, cell cycle and cell apoptosis in PCOS GCs and KGN cells

**Fig. 5:**
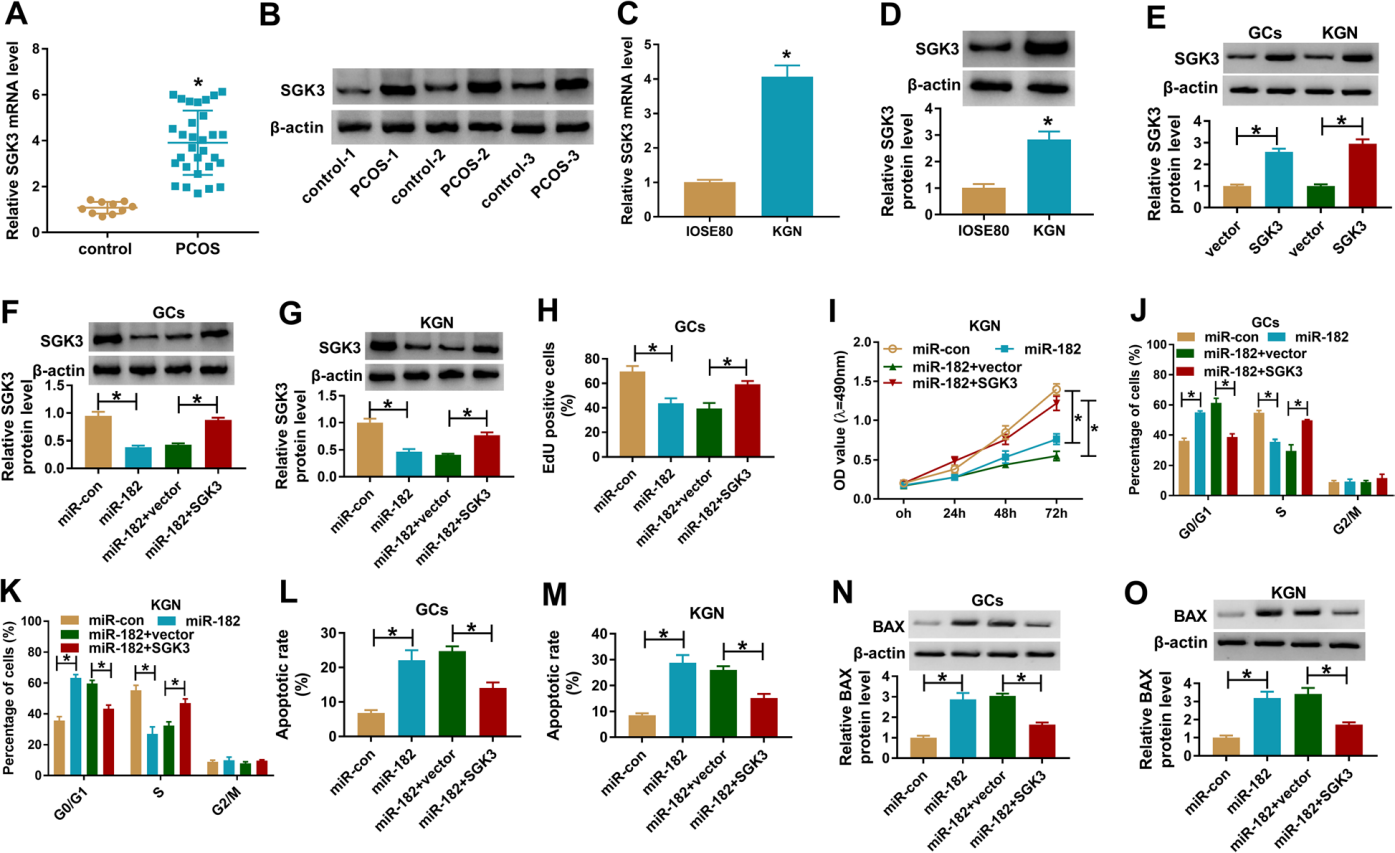
The effects of miR-182 on cell proliferation, cell cycle and cell apoptosis were abrogated by SGK3 overexpression in PCOS GCs and KGN cells. (**A** and **B**) The mRNA and protein levels of SGK3 in GCs from PCOS patients and normal controls were determined by qRT-PCR and western blot assay, respectively. (**C** and **D**) The mRNA and protein levels of SGK3 in IOSE80 and KGN cells were determined by qRT-PCR and western blot assay, respectively. (**E**) The protein level of SGK3 in PCOS GCs and KGN cells transfected with vector or SGK3 was measured through western blot assay. (**F**-**O**) PCOS GCs and KGN cells were divided into 4 groups: miR-con, miR-182, miR-182 + pcDNA and miR-182 + SGK3. (**F** and **G**) The mRNA and protein levels of SGK3 in transfected PCOS GCs and KGN cells were measured by qRT-PCR assay and western blot assay, respectively. (**H** and **I**) The proliferation of PCOS GCs and KGN cells was assessed by EdU assay and MTT assay. (**J**-**M**) Cell cycle and cell apoptosis in PCOS GCs and KGN cells were analyzed by flow cytometry analysis. (**N** and **O**) The protein level of BAX in PCOS GCs and KGN cells was examined with western blot assay.*P < 0.05

As presented in Fig. 5A and B, the mRNA and protein levels of SGK3 were markedly elevated in PCOS GCs compared to those in control groups (Fig. 5A and B). Furthermore, the mRNA and protein levels of SGK3 in IOSE80 and KGN cells were examined, showing that the mRNA and protein levels of SGK3 were conspicuously increased in KGN cells compared to IOSE80 cells (Fig. 5C and D). The overexpression vector of SGK3 transfection increased SGK2 protein level in both PCOS GCs and KGN cells compared to vector control groups (Fig. 5E). Afterward, to determine whether miR-182 could regulate the progression of PCOS by targeting SGK3, PCOS GCs and KGN cells were assigned to miR-con, miR-182, miR-182 + pcDNA and miR-182 + SGK3 groups. As demonstrated by western blot assay, miR-182 transfection markedly increased the protein level of SGK3 in PCOS GCs and KGN cells, while SGK3 transfection restored the effect (Fig. 5F and G). MTT assay proved that miR-182 elevation drastically inhibited the proliferation of PCOS GCs and KGN cells, while SGK3 overexpression restored the inhibitory effect (Fig. 5H and I). Flow cytometry analysis suggested that miR-182 induced cell cycle arrest in G0-G1 phase in PCOS GCs and KGN cells, but the administration of SGK3 weakened this impact (Fig. 5J and K). Moreover, the apoptosis of PCOS GCs and KGN cells was analyzed by flow cytometry analysis. The data exhibited that miR-182 facilitated cell apoptosis in PCOS GCs and KGN cells, whereas the upregulation of SGK3 partially restored the effect (Fig. 5L and M). Overexpression of miR-182 enhanced BAX protein level in PCOS GCs and KGN cells, while the impact was restored by increasing SGK3 (Fig. 5N and O). All these data demonstrated that miR-182 could suppress cell proliferation and promote cell cycle arrest and cell apoptosis by targeting SGK3 in PCOS GCs and KGN cells.

### Circ_0043532 positively regulated SGK3 expression by sponging miR-182

**Fig. 6:**
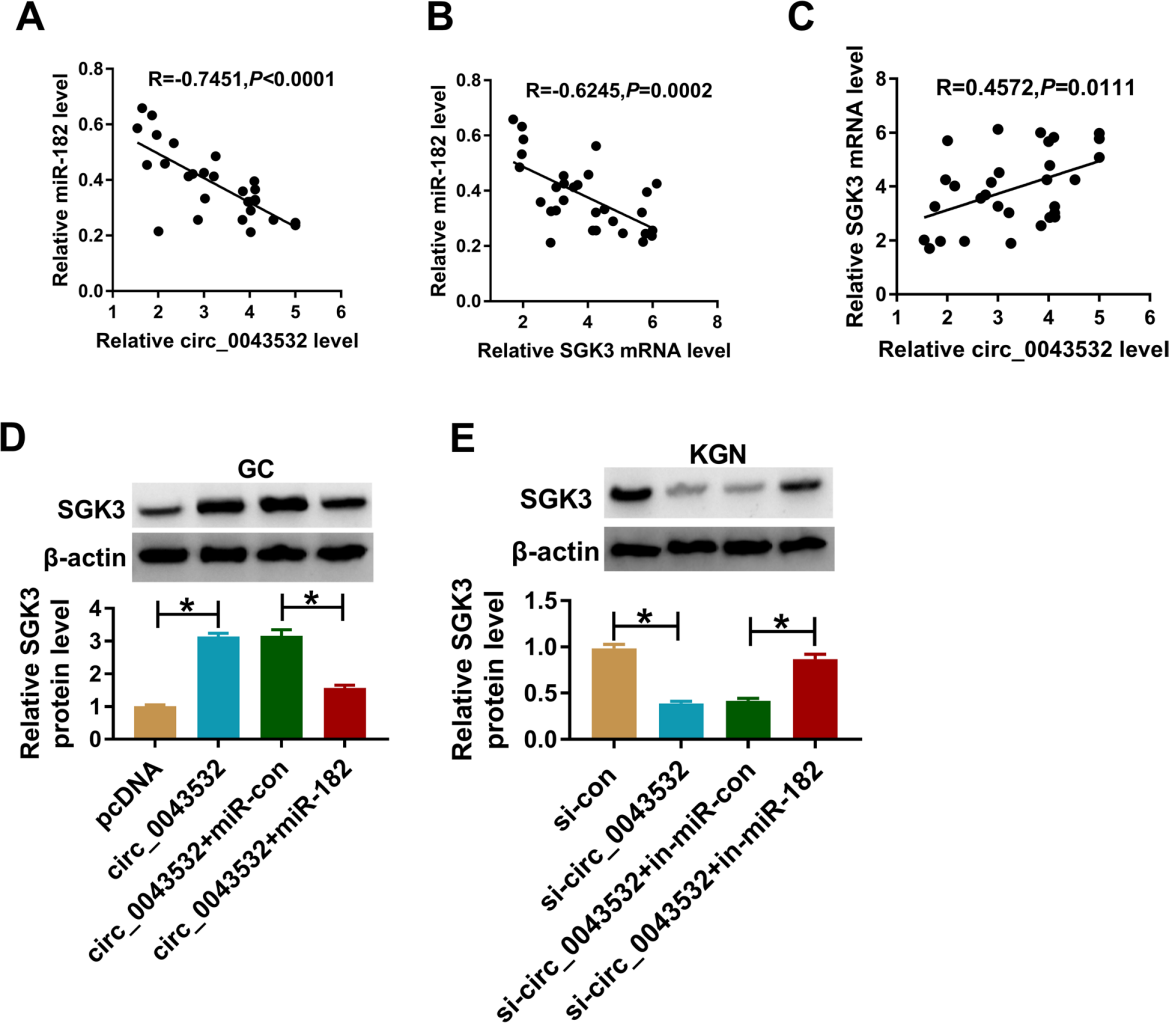
Circ_0043532 positively modulated SGK3 expression via sponging miR-182. (**A**-**C**) The correlations among circ_0043532, miR-182 and SGK3 in PCOS GCs were estimated by Spearman’s correlation coefficient analysis. (**D** and **E**) PCOS GCs and KGN cells were transfected with pcDNA, circ_0043532, circ_0043532 + miR-con, circ_0043532 + miR-182, si-con, si-circ_0043532, si-circ_0043532 + in-miR-con or si-circ_0043532 + in-miR-182, and then the protein level of SGK3 was measured by western blot assay. *P < 0.05

At last, we further investigated the relationships among circ_0043532, miR-182 and SGK3. The results of Spearman’s correlation coefficient analysis showed that miR-182 expression was inversely correlated with circ_0043532 or SGK3 mRNA expression in PCOS GCs (Fig. 6A and B). Moreover, we observed that there was a positive correlation between the levels of circ_0043532 and SGK3 mRNA in PCOS GCs (Fig. 6C). In addition, we found that circ_0043532 markedly enahanced the protein level of SGK3 in PCOS GCs, while miR-182 elevation overturned the effect (Fig. 6D). Circ_0043532 silencing reduced SGK3 protein level in KGN cells, with miR-182 inhibition reversed the effect (Fig. 6E). These results illustrated that circ_0043532 driectly targeted miR-182 to promote SGK3 expression in PCOS GCs and KGN cells.

## Discussion

Due to the increasing incidence of PCOS, it has received extensive attention from researchers [23]. Previous studies have shown that circRNAs are linked to the pathogenesis of PCOS [8]. The occurrence of PCOS was associated with ovarian GCs growth, survival and apoptosis [24, 25]. KGN cells have the majority of physiological characteristics of ovarian cells. Thus, GCs from patients with PCOS and KGN cells were used in this study. We found circ_0043532 expression was conspicuously raised in PCOS GCs and KGN cells. Circ_0043532 silencing repressed cell proliferation and promoted cell cycle arrest and cell apoptosis in PCOS GCs and KGN cells by regulation of miR-182/SGK3 axis.

Although some circRNAs have been demonstrated to be dysregulated in PCOS and take part in the regulation of PCOS progression [6, 7], the exact roles of circRNAs in PCOS development are largely unknown. Herein, we explored the effects and underlying mechanisms of circ_0043532 in PCOS development. Our results exhibited that circ_0043532 was aberrantly increased in PCOS GCs and KGN cells. Thus, we wondered if circ_0043532 played a role in PCOS. Thereafter, loss-of-function experiments were conducted. The results showed that silencing of circ_0043532 restrained cell growth and cell cycle and induced apoptosis in PCOS GCs and KGN cells, indicating that circ_0043532 knockdown could decelerate the progression of PCOS.

MiRNAs are related to the development of PCOS, as demonstrated by previous studies [26]. For example, Sun et al. suggested that miR-204 was declined in PCOS ovarian cortexes and KGN cells, and miR-204 upregulation hampered cell viability and facilitated cell apoptosis and cell cycle arrest in KGN cells [27]. He et al. declared that miR-200b and miR-200c were elevated in PCOS patients and their elevation could hamper the growth of KGN cells [28]. Geng et al. illustrated that miR-99a was weakly expressed in PCOS patients and the elevation of miR-99a could depress GCs growth and facilitate GCs apoptosis [29]. It is well documented that circRNAs can function as miRNA sponges to regulate the downstream target gene expression [30]. In the present study, miR-182 was identified as a target of circ_0043532. MiR-182 was involved in diverse human diseases, such as colorectal cancer (CRC) [31], laryngocarcinoma [32], HCC [33] non-small cell lung cancer [34]. Herein, we explored the effect of miR-182 in PCOS development for the first time. MiR-182 was weakly expressed in GCs from PCOS patients and KGN cells. MiR-182 inhibition effectively restored the impacts of circ_0043532 interference on cell proliferation, cell cycle and apoptosis in PCOS GCs and KGN cells. Furthermore, miR-182 upregulation could repress cell growth, induce cell cycle arrest and apoptosis in PCOS GCs and KGN cells.

Additionally, SGK3 was confirmed to be a target gene of miR-182. SGK3 was drastically increased in PCOS GCs and KGN cells, and there was a negative correlation between miR-182 and SGK3 in GCs from PCOS patients. Wu et al. manifested that SGK3 was increased in HCC and targeted by miR-144-3p to participate in the regulation of HCC [18]. Liu et al. claimed that SGK3 could function as a target of miR-212-3p and SGK3 silencing suppressed cell viability in glioblastoma [35]. Yao et al. demonstrated that SGK3 was targeted by miR-335-5p and SGK3 was increased in GCs from PCOS patients; furthermore, miR-335-5p suppressed GCs growth by targeting SGK3 [20]. Consistently, SGK3 silencing could hamper cell proliferase, cause cell cycle arrest and induce cell apoptosis in PCOS GCs and KGN cells. Moreover, SGK3 overexpression could abolish the effect of miR-182 overexpression on PCOS development, indicating that miR-182 participated in PCOS by targeting SGK3.

Stubbs et al. showed that GC mitosis and the proportion of preantral follicles with minichromosome maintenance protein 2 (MCM2)-positive GCs in anovulatory polycystic ovaries (anovPCO) were increased compared to normal ovulatory PCO (ovPCO) [25]. Das et al. indicated that cell apoptosis was reduced in PCOS [24]. However, whether circ_0043532 can regulate GC mitosis and apoptosis to influence the size of follicles is not clear, and it may be a interesting top in our futher study.

**Fig. 7:**
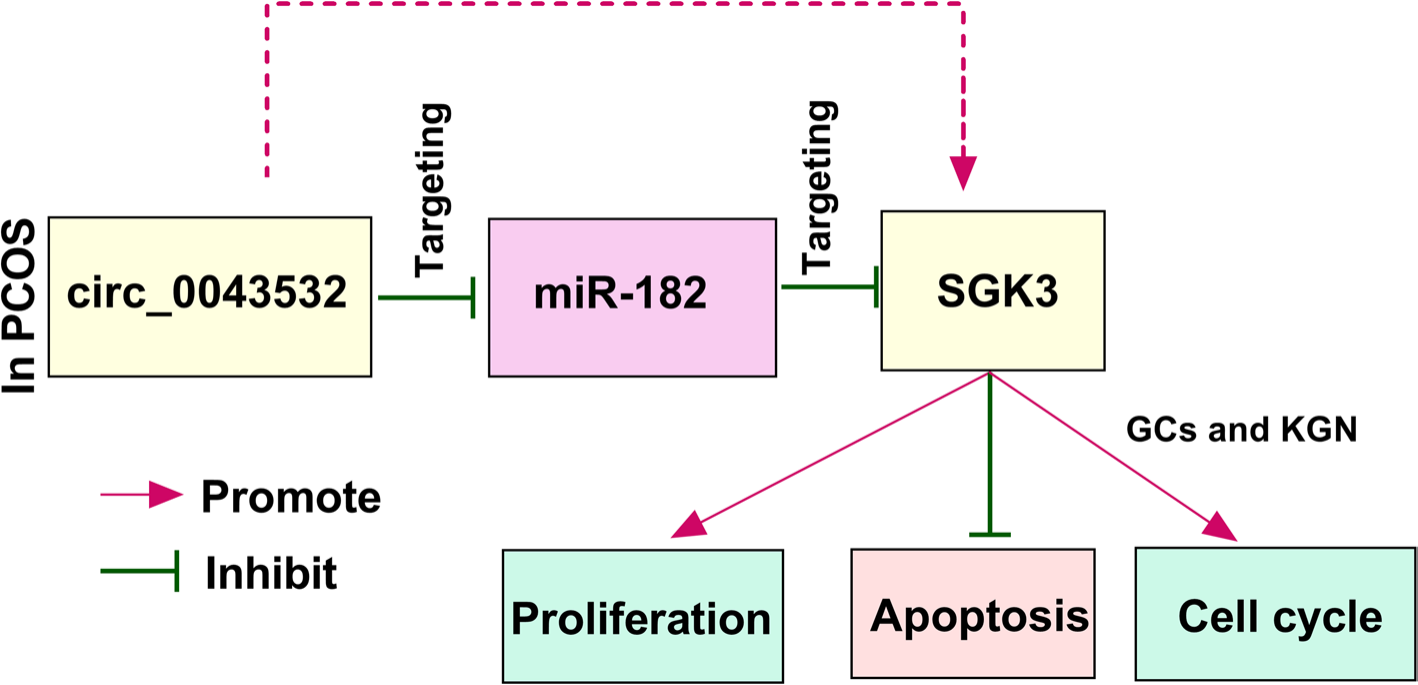
The schematic diagram of circ_0043532 in regulating cell proliferation, apoptosis and cell cycle

In conclusion, circ_0043532 was increased in PCOS GCs and KGN cells. Circ_0043532 promoted cell growth and cell cycle and suppressed cell apoptosis in PCOS GCs and KGN cells via modulating miR-182/SGK3 axis (Fig. 7). These findings might offer novel therapeutic targets for PCOS patients.

## Data Availability

The analyzed data sets generated during the present study are available from the corresponding author on reasonable request.

## Abbreviations

PCOS: Polycystic ovary syndrome
qRT-PCR: Quantitative real-time polymerase chain reaction
SGK3: Serum/glucocorticoid regulated kinase family member 3
RIP: RNA Immunoprecipitation
GCs: Granulosa cells
AGC: Aggrecan
OS: Osteosarcoma
HCC: Hepatocellular carcinoma
